# Supplementary material for: Translation, Adaptation and Assessment of the Psychometric Properties of the Mental Health Knowledge Questionnaire in a Sample of Higher Education Students in Portugal
Source: Int J Environ Res Public Health. 2023 Feb 9;20(4):3022. doi: 10.3390/ijerph20043022 (PMC9967354; doi:10.3390/ijerph20043022)
Supplement: Supplementary file 1 [file ijerph-20-03022-s001.zip › ijerph-2145038-supplementary.pdf]

Table S1 Questionário de Conhecimento sobre Saúde Mental

Item	Discordo Totalmente (1)	Discordo (2)	Não Concordo Nem Discordo (3)	Concordo (4)	Concordo Totalmente (5)
**Dimensão 1 – Conhecimento sobre as caraterísticas da saúde mental e dos distúrbios mentais.**					
Muitas pessoas têm problemas mentais, mas não se apercebem disso.					
Os componentes da saúde mental incluem inteligência normal, humor estável, atitude positiva, boas relações interpessoais, e capacidade de adaptação.					
Devem ser procurados serviços psicológicos ou psiquiátricos se suspeitarmos da presença de problemas ou distúrbios mentais.					
Atitudes positivas, boas relações interpessoais e um estilo de vida saudável podem ajudar a manter a saúde mental.					
Indivíduos com história familiar de distúrbios mentais correm maior risco de problemas psicológicos e distúrbios mentais.					
Problemas ou distúrbios mentais podem ocorrer quando um indivíduo está sob stress psicológico ou enfrenta um importante evento na sua vida (por exemplo, a morte de membros da família).					
**Dimensão 2 – Crença na epidemiologia dos distúrbios mentais.**					
Todos os distúrbios mentais são causados por stressores externos.					
Mesmo para distúrbios mentais graves (por exemplo, esquizofrenia) os medicamentos apenas devem ser tomados por um determinado período de tempo.					
Problemas psicológicos nos adolescentes não influenciam o seu desempenho escolar.					
É improvável que indivíduos de meia-idade ou idosos desenvolvam problemas psicológicos e distúrbios mentais.					
**Dimensão 3 – Consciencialização acerca de atividades de promoção da saúde mental.**					
Já ouviu falar sobre o Dia Mundial da Saúde Mental?					
Já ouvir falar sobre o Dia Internacional contra o Abuso e o Tráfico Ilícito de Drogas?					
Já ouviu falar sobre o Dia Mundial da Prevenção do Suicídio?					
Já ouviu falar sobre o Dia Mundial do Sono?					
